# Composite patient-reported outcomes and risk prediction for overall survival in advanced non-small cell lung cancer with first-line cemiplimab

**DOI:** 10.3389/fonc.2026.1676687

**Published:** 2026-02-25

**Authors:** David Gandara, Miranda Gogishvili, Ahmet Sezer, Tamta Makharadze, Mahmut Gümüş, Eric Yan, James Harnett, Ruben G. W. Quek

**Affiliations:** 1 Division of Hematology and Oncology, Department of Medicine, UC Davis Comprehensive Cancer Center, Sacramento, CA, United States; 2 High Technology Medical Centre, University Clinic Ltd, Tbilisi, Georgia; 3 Department of Medical Oncology, Başkent University, Adana, Türkiye; 4 LTD High Technology Hospital Med Center, Batumi, Georgia; 5 Department of Medical Oncology, School of Medicine, Istanbul Medeniyet University, Istanbul, Türkiye; 6 Cyan Global Inc., San Diego, CA, United States; 7 Regeneron Pharmaceuticals, Inc., Tarrytown, NY, United States

**Keywords:** cemiplimab, immunotherapy, non-small cell lung cancer, patient-reported outcomes, PD-1

## Abstract

**Introduction:**

Patient-reported outcomes (PROs) are associated with overall survival (OS) in advanced cancer. Risk modeling of OS based on a single PRO scale was previously evaluated in patients with advanced non-small cell lung cancer (NSCLC) in two pivotal cemiplimab ± chemotherapy phase III trials. Here we report evaluation of predictive performance for a composite of two PRO scales.

**Methods:**

Data from two previously published phase III clinical trials (EMPOWER-Lung 1 and EMPOWER-Lung 3) were used to develop a Cox proportional hazards model evaluating the association between baseline PRO burden and OS, stratified by treatment, histology, and programmed cell death-ligand 1 (PD-L1) level. PRO data were collected using the European Organisation for Research and Treatment of Cancer (EORTC) Quality of Life Questionnaire Core 30 (QLQ-C30) and Lung Cancer 13 (QLQ-LC13) modules. Single-scale PROs and composite PROs based on a combination of one functioning and one symptom scale were evaluated for prognostic value for OS using hazard ratios (HRs; a higher HR indicates a higher risk of death).

**Results:**

The top 10 composite PROs that predicted the highest risk for death (nominal *P* values <.05 and HRs ≥2) included combinations of functioning scales (ie, social, role, and physical) and select symptom scales (ie, dyspnea, appetite loss, and pain from the EORTC QLQ-C30; dyspnea and coughing from the EORTC QLQ-LC13). Patients with composite PROs of low functioning and high symptom burden had worse OS than those with high functioning and low symptom burden. A clear separation in Kaplan–Meier survival curves was observed between high-risk and low-risk groups based on the composite PRO measure of role functioning and dyspnea.

**Discussion:**

In patients with advanced NSCLC receiving first-line cemiplimab-based therapy, composite PROs consisting of one functioning and one symptom scale had greater prognostic value than single PRO scales. Further development and analysis of composite PROs for clinical trials is warranted.

## Introduction

Studies evaluating novel therapies for the treatment of cancer typically focus on tumor response, progression-free survival (PFS), and overall survival (OS) outcomes. However, patient-reported outcomes (PROs) are increasingly recognized as equally important indicators of prognosis and efficacy outcomes (1). A number of PRO risk models for OS in cancer have previously been described (1–3). However, PRO risk modeling for patients receiving immune checkpoint inhibitors (ICIs) has been less well established; furthermore, there are no published data on the prognostic value of a composite measure of PROs for patients receiving ICIs (3, 4).

The most commonly used PRO instruments in non-small cell lung cancer (NSCLC) clinical trials of ICIs include the cancer-specific European Organisation for Research and Treatment of Cancer Quality of Life Questionnaire Core 30 (EORTC QLQ-C30), generic health EuroQol 5 Dimension Scale, and disease-specific instruments such as the EORTC QLQ Lung Cancer 13 (LC13) module (5).

**Figure 1:**
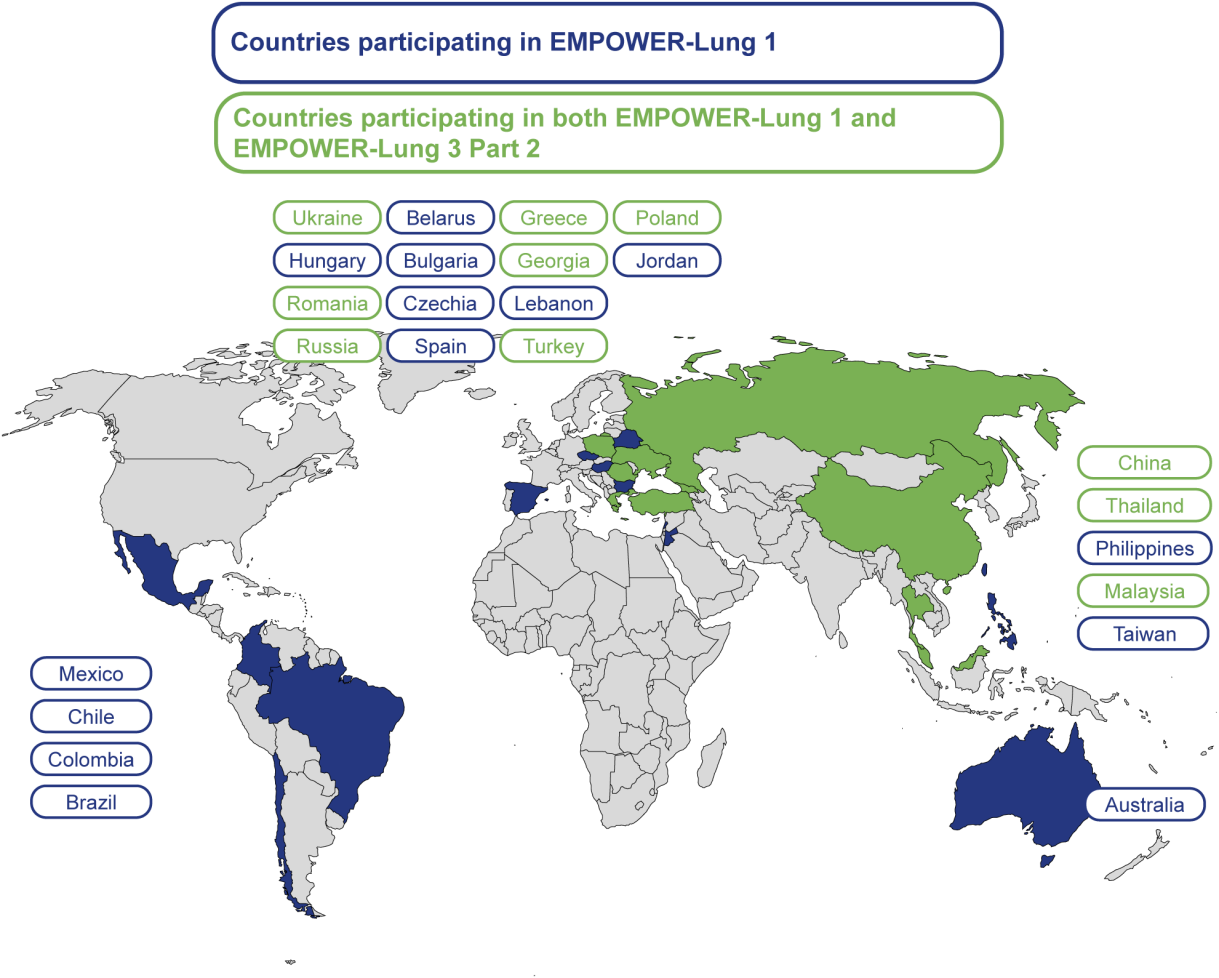
Participating countries for the EMPOWER-Lung 1 and EMPOWER-Lung 3 clinical trials.

Cemiplimab (a programmed cell death-1 inhibitor) is approved in various countries for first-line treatment in adult patients with advanced non-small cell lung cancer (aNSCLC) with no epidermal growth factor receptor (*EGFR*), anaplastic lymphoma kinase (*ALK*), or c-ros oncogene 1 (*ROS1*) genomic aberrations (6–8). Cemiplimab is approved by the United States Food and Drug Administration (FDA) for those with aNSCLC, regardless of histology, as a monotherapy for those with programmed cell death-ligand 1 (PD-L1) expression level of ≥50%, as well as in combination with platinum-based chemotherapy regardless of PD-L1 levels (6). The approvals were based on published data from EMPOWER-Lung 1 (NCT03088540) and EMPOWER-Lung 3 Part 2 (NCT03409614), two pivotal phase III trials that enrolled patients from various countries around the world (**Figure 1**) (9–11).

EMPOWER-Lung 1 is a multicenter, open-label, randomized study of patients with aNSCLC with PD-L1 expression of ≥50%, comparing cemiplimab monotherapy (n=283) with platinum-doublet chemotherapy (n=280) in the first-line treatment setting (11). Results from this study showed improvements in survival and PROs associated with cemiplimab treatment versus platinum-doublet chemotherapy (11, 12). EMPOWER-Lung 3 Part 2 compared the efficacy and safety of cemiplimab plus chemotherapy (n=312) versus chemotherapy alone (n=154) in patients with aNSCLC with no *EGFR*, *ALK*, or *ROS1* genomic aberrations and irrespective of PD-L1 expression (10, 13). Consistent with findings from EMPOWER-Lung 1, results from this study showed benefits in both survival and PROs with cemiplimab treatment, this time in combination with chemotherapy, versus chemotherapy alone.

The objective of the current study was to identify and develop composite PRO measures that ascertain risk of death in patients with aNSCLC receiving first-line cemiplimab treatment, using data from EMPOWER-Lung 1 and EMPOWER-Lung 3. The intent of developing composite scales was to capture multi-dimensional patient states. This approach could lead to a prioritized set of composite PROs to be incorporated in a simple clinical practice tool that may assist clinicians in better understanding risk of death, informing decision-making about the need for additional monitoring/support of high-risk patients. Such a tool may be used in conjunction with innovative payment models such as the Enhancing Oncology model, which requires collection of PROs (14).

## Materials and methods

### Study design and patients

This study utilized PRO data that were collected from two previously reported phase III clinical studies: EMPOWER-Lung 1 (cemiplimab monotherapy treatment arm [n=283]) and EMPOWER-Lung 3 Part 2 (cemiplimab + chemotherapy treatment arm [n=312]). The study designs are shown in **Supplementary Table S1** (10, 11).

Both studies enrolled patients aged ≥18 years with histologically or cytologically confirmed squamous or non-squamous stage IIIB/C (if deemed not candidates for treatment with definitive chemoradiation) or stage IV NSCLC, and an Eastern Cooperative Oncology Group (ECOG) performance status of ≤1 (11, 13). Study designs for the individual studies were previously reported: EMPOWER-Lung 1 enrolled patients with PD-L1 expression in ≥50% of tumor cells (11); EMPOWER-Lung 3 Part 2 enrolled patients with any level of PD-L1 expression (13).

PRO baseline data were collected using EORTC QLQ-C30 and -LC13 questionnaires (15, 16). The complete list of scales included in our analyses were: EORTC QLQ-C30 global health status/QoL; EORTC QLQ-C30 Functional Scales: physical functioning, role functioning, emotional functioning, cognitive functioning, and social functioning; EORTC QLQ-C30 Symptom Scales: fatigue, nausea and vomiting, pain, dyspnea, insomnia, appetite loss, and diarrhea; EORTC QLQ-LC13 Symptom Scales: cough, hemoptysis, dyspnea, pain in chest, pain in arm or shoulder, pain in other parts, sore mouth, peripheral neuropathy, and alopecia. For a single PRO scale, patients were divided into high- and low-risk burden groups based on their baseline PRO values using the median of NSCLC reference values (17) as the cut-points (**Table 1**). Composite PRO measures were constructed from the combination of one functioning scale and one symptom scale. The risk burden from a composite PRO measure is defined in **Table 2**.

**Table 1:** Risk burden derived from a single PRO scale.

Scales	PRO score	
	≤ median	> median
Functioning scales and GHS/QoL	High PRO burden	Low PRO burden
Symptom scales	Low PRO burden	High PRO burden

For functioning scales and GHS/QoL, patients who have baseline less than or equal to the median calculated from NSCLC reference values (17) are considered to have high risk burden; patients who have baseline greater than median are considered to have low risk burden.

For symptom scales, patients who have baseline less than or equal to median (17) are considered to have low risk burden; patients who have baseline greater than median are considered to have high risk burden.

GHS, global health status; NSCLC, non-small cell lung cancer; PRO, patient-reported outcome; QoL, quality of life.

**Table 2:** Risk burden derived from a baseline composite PRO measure.

Functioning scale burden	Symptom scale burden: Low	Symptom scale burden: High
Functioning scale burden: Low	Composite PRO burden: Low	Composite PRO burden: Medium
Functioning scale burden: High	Composite PRO burden: Medium	Composite PRO burden: High

Composite risk burden is low if both functioning burden and symptom burden from single PRO scales are low. Composite risk burden is high if both functioning burden and symptom burden from single PRO scales are high. For other combinations of functioning burden and symptom burden from single PRO scales, the composite risk burden is defined as medium.

PRO, patient-reported outcome.

### Statistical analysis

Cox proportional hazards models stratified by treatment, histology, and PD-L1 level were performed to evaluate the association between baseline PRO burden and OS. Single-scale PROs and composite PROs (based on the combination of one functioning and one symptom scale) were assessed for prognostic value for risk of death using hazard ratios (HRs). PROs with higher HRs when comparing high versus low PRO burden were deemed to have a higher risk of death. Kaplan-Meier curves were used to estimate the distribution of OS for composite measures.

## Results

### Baseline characteristics

Baseline characteristics of patients in EMPOWER-Lung 1 and EMPOWER-Lung 3 Part 2 are shown in **Supplementary Table S2**. Patient demographics and baseline clinical characteristics were generally similar between the two groups.

### Primary outcomes

**Figure 2:**
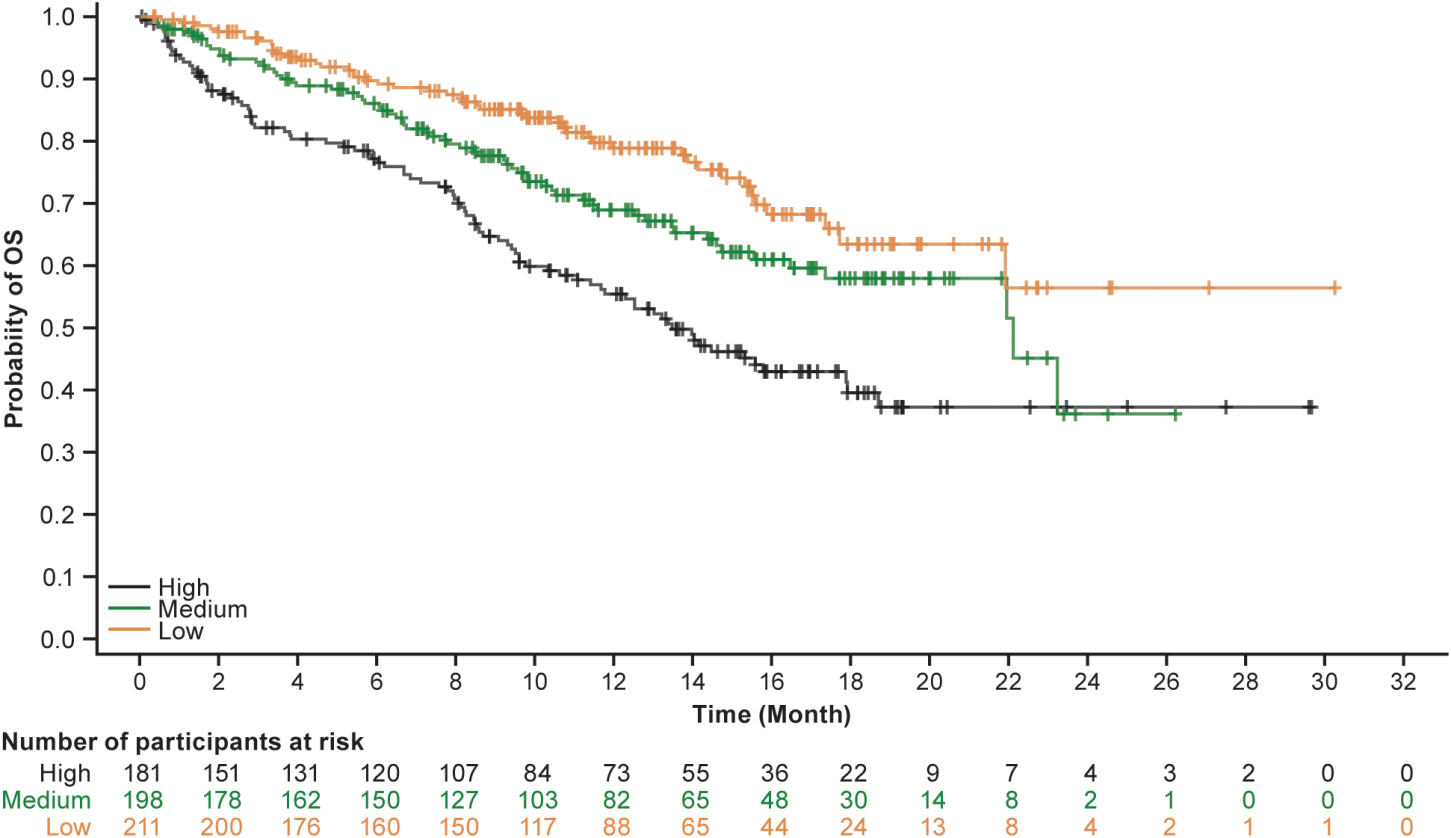
An example of a Kaplan-Meier plot: estimate of OS by risk of role functioning and LC-dyspnea. LC, lung cancer; OS, overall survival.

The top 10 composite PROs that predicted the highest risk for death had nominal *P* values <.05 and HRs ≥2 (**Table 3**). The top 10 single PROs that predicted the highest risk for death had nominal *P* values <.05 but all HRs <2 (**Table 3**). That is, all top 10 composite PROs had higher HRs than any single PRO. The top 10 composite PROs included functioning scales of social, role, and physical; and symptom scales of dyspnea, appetite loss, and pain from the EORTC QLQ-C30; and dyspnea and coughing from the EORTC QLQ-LC13. Patients with composite PROs of low functioning and high symptom burden (the high-risk group) had worse OS than those with high functioning and low symptom burden (the low-risk group). Three composite PRO measures (social functioning and lung cancer [LC]-dyspnea, social functioning and dyspnea, and role functioning and LC-dyspnea) had the highest prognostic value. The Kaplan-Meier curves associated with these three composite PRO measures showed clear separation between high-risk and low-risk groups. For example, the Kaplan-Meier curve estimating OS by the role functioning and LC-dyspnea composite PRO is shown in **Figure 2**.

**Table 3:** Top 10 overall survival HRs of composite and single PROs at baseline from a Cox proportional model.

PROs	Patients at risk, n	HR (95% CI)	*P* value
Composite PRO			
SF*LC-dyspnea	591	2.52 (1.75–3.64)	<.001
SF*dyspnea	592	2.48 (1.67–3.69)	<.001
RF*LC-dyspnea	590	2.42 (1.69–3.48)	<.001
SF*appetite loss	592	2.42 (1.66–3.55)	<.001
RF*appetite loss	592	2.40 (1.63–3.55)	<.001
PF*appetite loss	592	2.37 (1.59–3.53)	<.001
SF*LC-coughing	592	2.35 (1.57–3.54)	<.001
SF*pain	593	2.32 (1.60–3.37)	<.001
RF*dyspnea	592	2.32 (1.56–3.46)	<.001
PF*pain	592	2.27 (1.54–3.35)	<.001
Single PRO^¶^			
SF	593	1.92 (1.43–2.58)	<.001
PF	592	1.88 (1.36–2.59)	<.001
RF	592	1.88 (1.39–2.53)	<.001
LC-dyspnea	591	1.76 (1.32–2.34)	<.001
Fatigue	592	1.58 (1.19–2.09)	.002
Dyspnea	592	1.51 (1.10–2.08)	.011
Pain	593	1.50 (1.13–2.00)	.005
Appetite loss	592	1.50 (1.13–2.00)	.005
GHS/QoL	593	1.39 (1.05–1.84)	.023
LC-coughing	592	1.37 (1.02–1.85)	.037

LC-dysphagia and constipation were excluded *a priori* from the analyses as they were not considered relevant symptoms among patients with aNSCLC receiving immunotherapy ± chemotherapy.

^¶^The complete list of scales included in our analyses were: EORTC QLQ-C30 global health status/QoL; EORTC QLQ-C30 Functional Scales: physical functioning, role functioning, emotional functioning, cognitive functioning, and social functioning; EORTC QLQ-C30 Symptom Scales: fatigue, nausea and vomiting, pain, dyspnea, insomnia, appetite loss, and diarrhea; EORTC QLQ-LC13 Symptom Scales: cough, hemoptysis, dyspnea, pain in chest, pain in arm or shoulder, pain in other parts, sore mouth, peripheral neuropathy, and alopecia.

aNSCLC, advanced non-small cell lung cancer; C30, Core 30; EORTC QLQ, European Organisation for Research and Treatment of Cancer Quality of Life Questionnaire; GHS, global health status; LC, lung cancer; LC13, Lung Cancer 13; PF, physical functioning; PRO, patient-reported outcome; QoL, quality of life; RF, role functioning; SF, social functioning.

## Discussion

To our knowledge, this is the first composite PRO analysis of patients with aNSCLC receiving first-line checkpoint immunotherapy. PROs in aNSCLC have previously been examined for first-line cemiplimab (as monotherapy and in combination with chemotherapy) in phase III trials (12, 18); overall improvement in symptoms and delayed time to definitive clinically meaningful deterioration in cancer-related and lung cancer-specific symptoms and functions were observed. Prior research has also shown that single-scale PROs were prognostic for survival outcomes in patients with aNSCLC who initiated first-line cemiplimab-based therapy (19). This current report showed that a risk model of OS using baseline PROs demonstrated greater prognostic value with composite PROs (consisting of a functioning and a symptom scale) compared to relying on single PRO scales. Predictive utility of composite PROs for evaluating the risk of death would undoubtedly have value for patients and investigators.

We developed a risk model of OS using baseline PROs in patients with aNSCLC treated with first-line cemiplimab-based therapy. Patients in the high-risk group (low functioning and high symptom burden) had a lower probability of survival than those in the low-risk group (high functioning and low symptom burden). Our results are in line with previous research that found prognostic value of PROs, specifically physical functioning, in patients with lung cancer (1).

Regulatory bodies such as the FDA have acknowledged the added value of incorporating PRO measurements of symptoms and functional impacts into the benefit/risk assessment of clinical trials. A core set of PROs that may be important contributors to a patient’s health-related quality of life and that may be sensitive to effects of disease and treatment has been previously explored (20). Recent guidance from the FDA recommends collection of specific core PROs in cancer clinical trials, including physical functioning, role functioning, and disease-related symptoms (21). In our study, the top 10 composite PROs that predicted the highest risk for death included combinations of functioning scales (ie, social, role, and physical) and select disease-related symptom scales (ie, dyspnea, appetite loss, and pain from the EORTC QLQ-C30; dyspnea and coughing from the EORTC QLQ-LC13). These may guide clinicians regarding the prioritization, selection, and inclusion of PROs to minimize patient burden and improve the quality of data collected by focusing on the most meaningful and measurable outcomes associated with OS in aNSCLC trials. Prioritization of composite PROs with the highest prognostic value for OS may potentially help reduce the heterogeneity in PRO assessment strategies and potentially increase the regulatory utility of PRO data from aNSCLC trials. In clinical practice, if the composite PROs with the highest prognostic value for OS are collected, treating physicians will have a greater ability to predict patients’ OS and therefore provide better treatment care to patients accordingly.

Furthermore, a risk model that utilizes PRO data to estimate survival could be useful in the clinical setting in the context of more patient-centered healthcare models such as the Enhancing Oncology model. The Enhancing Oncology model requires collection of electronic PRO data to monitor patient needs and guide treatment (14). This patient involvement facilitates better communication between patients and their treatment teams, which increases patients’ understanding of their symptoms and care and better informs treatment decisions (14). Guidelines provided by the Centers for Medicare & Medicaid Services recommend surveys that include symptom, functioning, and behavioral scales, such as the EORTC quality of life questionnaires (14). A composite PRO tool could be used as part of these patient-centered healthcare models, either as an option for PRO data collection or as part of a digital PRO dashboard that is used to assess individual patient cases.

Our study’s systematic assessment identified a prioritized fit-for-purpose set of composite PROs associated with the highest risk for death; these may facilitate high-quality data collection of patient-reported symptoms and functional impacts among cancer patients. Although the results of this study provide valuable insight, there are limitations to keep in mind. As this was an exploratory and hypothesis-generating analysis, findings need to be validated by further research in an independent cohort. For example, PROs collected from patients with aNSCLC in a real-world setting would provide valuable data. This analysis only included data from a specific patient population and results may be limited to patients with similar characteristics (eg, indication and PD-L1 expression of ≥50%) and may not be generalizable to other tumor and treatment types. The potential for overfitting, given the number of composite combinations that were tested, and use of nominal *P* values (without adjustment for multiple testing) should also be held in consideration. Further development and analysis of composite PROs for clinical trials, including validation studies, may be warranted.

## Conclusions

This research shows that PROs have prognostic value for OS in patients with aNSCLC who received first-line cemiplimab therapy. These data contribute to a growing niche of literature on the value of PROs beyond clinical trial endpoints. Using baseline composite PROs of one functioning scale and one symptom scale to evaluate OS risk in aNSCLC could help inform clinicians and patients on the most appropriate treatment pathway and support shared decision-making.

## Data Availability

Qualified researchers may request access to study documents (including the clinical study report, study protocol with any amendments, blank case report form, and statistical analysis plan) that support the methods and findings reported in this manuscript. Individual anonymized participant data will be considered for sharing once the product and indication has been approved by major health authorities (eg, US Food and Drug Administration, European Medicines Agency, Pharmaceuticals and Medical Devices Agency, etc.), if there is legal authority to share the data and there is not a reasonable likelihood of participant re identification. Requests to access the datasets should be directed to https://vivli.org/.
