# Supplementary material for: Composite patient-reported outcomes and risk prediction for overall survival in advanced non-small cell lung cancer with first-line cemiplimab
Source: Front Oncol. 2026 Feb 25;16:1676687. doi: 10.3389/fonc.2026.1676687 (PMC12975601; doi:10.3389/fonc.2026.1676687)
Supplement: Supplementary file 1 [file Table1.docx]

Supplementary Table S1. Study designs for (A) EMPOWER-Lung 1 and (B) EMPOWER-Lung 3 (Part 2)

|  | EMPOWER-Lung 1^1^ | EMPOWER-Lung 3 Part 2^2^ |
| --- | --- | --- |
| **NCT identifier** | NCT03088540 | NCT03409614 |
| **Patient population** | Advanced or metastatic NSCLC with PD-L1 expression ≥50% | Advanced or metastatic NSCLC and any level of PD-L1 expression |
| **Treatment arms** | 1:1 randomization  **Arm A**  Cemiplimab 350 mg IV Q3W treatment for 108 weeks or until disease progression  After disease progression, patients were given the option to continue cemiplimab + 4 cycles of chemotherapy  **Arm B**  4–6 cycles of investigator’s choice of chemotherapy  After disease progression, patients were given the option to crossover to cemiplimab monotherapy | 2:1 randomization  **Arm A**  Cemiplimab 350 mg IV Q3W + investigator’s choice of platinum doublet chemotherapy for 4 cycles^†^  **Arm B**  Placebo Q3W + investigator’s choice of platinum doublet chemotherapy for 4 cycles^†^ |

Abbreviations: IV, intravenous; NSCLC, non-small cell lung cancer; PD-L1, programmed cell death-ligand 1; Q3W, every 3 weeks.

^†^From randomization to data cutoff date.

Supplementary Table S2. Baseline characteristics of patients in the two treatment arms in EMPOWER-Lung 1 and EMPOWER-Lung 3 Part 2

|  | **Cemiplimab (n=283)^1^** | **Cemiplimab + chemotherapy (n=312)^3^** | **Overall (N=595)** |
| --- | --- | --- | --- |
| Age, median (IQR), years | 63 (58–69) | 63 (57–68) | 63 (57–68) |
| ≥65 years, n (%) | 126 (45) | 128 (41) | 254 (43) |
| Sex, n (%) |  |  |  |
| Male | 248 (88) | 268 (86) | 516 (87) |
| Female | 35 (12) | 44 (14) | 79 (13) |
| Geographic region, n (%) | | | |
| Europe | 215 (76) | 270 (87) | 485 (82) |
| Asia | 31 (11) | 42 (14) | 73 (12) |
| Rest of the world | 37 (13) | 0 | 37 (6) |
| ECOG PS, n (%) | | | |
| 0 | 77 (27) | 51 (16) | 128 (22) |
| 1 | 206 (73) | 259 (83) | 465 (78) |
| Cancer stage at screening, n (%) | | | |
| Locally advanced | 45 (16) | 45 (14) | 90 (15) |
| Metastatic | 238 (84) | 267 (86) | 505 (85) |

Abbreviations: ECOG PS, Eastern Cooperative Oncology Group performance status; IQR, interquartile range.

# References

1. Sezer A, Kilickap S, Gümüş M, et al. Cemiplimab monotherapy for first-line treatment of advanced non-small-cell lung cancer with PD-L1 of at least 50%: A multicentre, open-label, global, phase 3, randomised, controlled trial. *Lancet*. 2021;397(10274):592–604.

2. Makharadze T, Gogishvili M, Melkadze T, et al. Cemiplimab plus chemotherapy versus chemotherapy alone in advanced NSCLC: 2-year follow-up from the phase 3 EMPOWER-Lung 3 Part 2 trial. *J Thorac Oncol*. 2023;18(6):755–768.

3. Gogishvili M, Melkadze T, Makharadze T, et al. Cemiplimab plus chemotherapy versus chemotherapy alone in non-small cell lung cancer: A randomized, controlled, double-blind phase 3 trial. *Nat Med*. 2022;28:2374–2380.
